# Modified arthroscopic intra-articular transtendinous looped biceps tenodesis leads to satisfactory functional outcomes and less frequent Popeye deformity compared to biceps tenotomy

**DOI:** 10.1186/s13018-023-04078-9

**Published:** 2023-08-16

**Authors:** Chen-Hao Chiang, Wei Ho, Chang-Hao Lin, Wei-Hsing Chih, Wei-Ren Su, Chih-Kai Hong

**Affiliations:** 1 Department of Orthopaedics, Ditmanson Medical Foundation Chia-Yi Christian Hospital, Chiayi, Taiwan; 2 Department of Orthopaedic Surgery, National Cheng Kung University Hospital, College of Medicine, National Cheng Kung University, No.138, Sheng-Li Road, North Dist., Tainan, 70428 Taiwan; 3 Skeleton Materials and Bio-Compatibility Core Lab, Research Center of Clinical Medicine, National Cheng Kung University Hospital, College of Medicine, National Cheng Kung University, Tainan, Taiwan; 4 Musculoskeletal Research Center, Innovation Headquarter, National Cheng Kung University, Tainan, Taiwan; 5 Department of Orthopaedic Surgery, National Cheng Kung University Hospital, Dou-Liou Branch, Yunlin, Taiwan

**Keywords:** Long head of biceps tendon, Tenotomy, Tenodesis, Popeye deformity

## Abstract

**Purpose:**

The present study aimed to propose a modified intra-articular transtendinous looped biceps tenodesis (mTLBT) using a suture anchor and to compare the functional outcomes and incidence of Popeye deformities between biceps tenotomy and mTLBT.

**Methods:**

Medical records of patients who underwent either tenotomy or mTLBT for the long head of the biceps tendon (LHBT) lesion between January 2016 and April 2021 were retrospectively reviewed. The inclusion criteria were patients aged 40–70 years with LHBT pathologies, such as superior labrum anterior to posterior (SLAP) lesions > type II, LHBT pulley system rupture with bicipital instability, and intra-articular LHBT tear. The exclusion criteria were full-thickness supraspinatus tears, frozen shoulder, shoulder fracture, and postoperative traumatic events that affected the operated shoulder. All patients were followed up for at least 1 year. Popeye deformity, bicipital cramping pain, visual analog scale (VAS) pain score, and functional outcome scores (University of California at Los Angeles [UCLA] and American Shoulder and Elbow Surgeons [ASES] scores) were recorded. Fisher’s exact test and Chi-square test were used for categorical variables, whereas the Mann–Whitney U test was used for nonparametric variables.

**Results:**

The mTLBT and tenotomy groups included 15 and 40 patients, respectively. The incidence of Popeye deformity and biceps cramping pain in the tenotomy group (52.5% and 50%, respectively) was significantly higher than that in the mTLBT group (13.3% and 20%, respectively) (*p* = 0.009 and *p* = 0.045, respectively). The postoperative VAS, UCLA, and ASES scores were not significantly different between the two groups. One patient in the tenodesis group experienced metallic-anchor pullout.

**Conclusion:**

mTLBT is an arthroscopic intra-articular top of the groove tenodesis that can be performed completely in the intra-articular space and is especially suitable for patients with an intact or partially torn rotator cuff. This technique is reliable for treating biceps pathologies as it results in similar functional outcome scores, lesser biceps cramping pain, and less frequent Popeye deformity compared to biceps tenotomy.

**Level of Evidence:**

III.

## Introduction

Tendinopathy of the long head of the biceps tendon (LHBT), which occurs in isolation or is accompanied by other shoulder pathologies, is a common cause of anterior shoulder pain [2, 28]. Surgical interventions for LHBT tendinopathy include tenotomy and tenodesis [13, 21, 28]. Although both tenotomy and tenodesis lead to comparable clinical outcomes [1, 21, 22, 24], there is a trend that patients prefer tenodesis to tenotomy owing to concerns regarding Popeye deformity [14, 15].

Numerous surgical techniques have been proposed for biceps tenodesis [10–12]. Some surgeons prefer suprapectoral biceps tenodesis as it can be performed arthroscopically [6, 20], whereas others prefer open subpectoral tenodesis because the surgical procedure is simple and allows treatment of lesions located in the bicipital groove [9, 19]. Among the techniques for arthroscopic suprapectoral tenodesis, some surgeons favor the transtendinous technique for tenodesis as it helps suture the tendon in arthroscopy surgery [16, 25].

A disadvantage of the currently practiced transtendinous biceps tenodesis techniques [16, 25] is that the procedure needs to be performed in both the subacromial and intra-articular spaces. For patients with intact or partially torn rotator cuffs, surgeons need to perform fixation of the LHBT in the subacromial space and tenotomy at the insertion site of the LHBT in the intra-articular space, thereby increasing the complexity of the surgical procedure.

To simplify the procedure of arthroscopic suprapectoral tenodesis, we developed a modified top of the groove tenodesis, named intra-articular transtendinous looped biceps tenodesis (mTLBT) technique. The technique can be performed completely in the intra-articular space and is especially suitable for patients with intact or partially torn rotator cuffs. The purpose of this study was to introduce an mTLBT technique using a suture anchor and to compare the functional outcomes and Popeye deformities between biceps tenotomy and mTLBT. Our hypothesis was that patients undergoing mTLBT would have lesser Popeye deformity and comparable functional outcomes to those undergoing biceps tenotomy.

## Methods

### Study population

The study was approved by the institutional review board (IRB No: 2022045) of the authors’ institute. Data of patients who underwent shoulder arthroscopy between January 2016 and April 2021 were retrospectively reviewed. The inclusion criteria were patients aged 40–70 years with LHBT pathologies, such as superior labrum anterior to posterior (SLAP) lesions > type II, LHBT pulley system rupture with bicipital instability, and intra-articular LHBT tear. Patients with SLAP lesions were included as both biceps tenotomy and tenodesis were reasonable treatment choices [30–32]. The exclusion criteria were full-thickness supraspinatus tears, LHBT tear distal to the proximal bicipital groove, medial dislodgement of the LHBT from the bicipital groove, frozen shoulder, glenoid fracture, proximal humeral fracture, advanced glenohumeral osteoarthritis, septic arthritis, and postoperative traumatic events that affected the operated shoulder. All patients were the followed-up for at least 1 year.

### Surgical technique

Surgical treatment procedures for LHBT pathologies varied in different periods. Between January 2016 and April 2019, LHBT pathologies were treated with biceps tenotomy, whereas mTLBTs were performed from May 2019 to April 2021.

All patients were treated by a single shoulder surgeon (C-H C). The operation was performed in a standard beach-chair position with the shoulder in 30° abduction and 10° flexion and the elbow in 90° flexion in an arm holder. The crucial landmarks of the shoulder and anticipated port insertion sites were carefully palpated and marked. The standard posterior portal, 2 cm inferior and 1 cm medial to the posterior acromial corner, was used as the view portal. Once LHBT pathologies or SLAP lesions were confirmed, the patients underwent either biceps tenotomy or mTLBT. A standard anterior portal was established for patients in the tenotomy group, whereas the anterosuperior and anteroinferior portals were used for patients undergoing mTLBT. The anteroinferior portal was set up near the superior border of the subscapularis, close to its tendon insertion. The anterosuperior portal was set up at the level of the inlet of the bicipital groove just anterior to the anterior border of the supraspinatus.

**Fig. 1:**
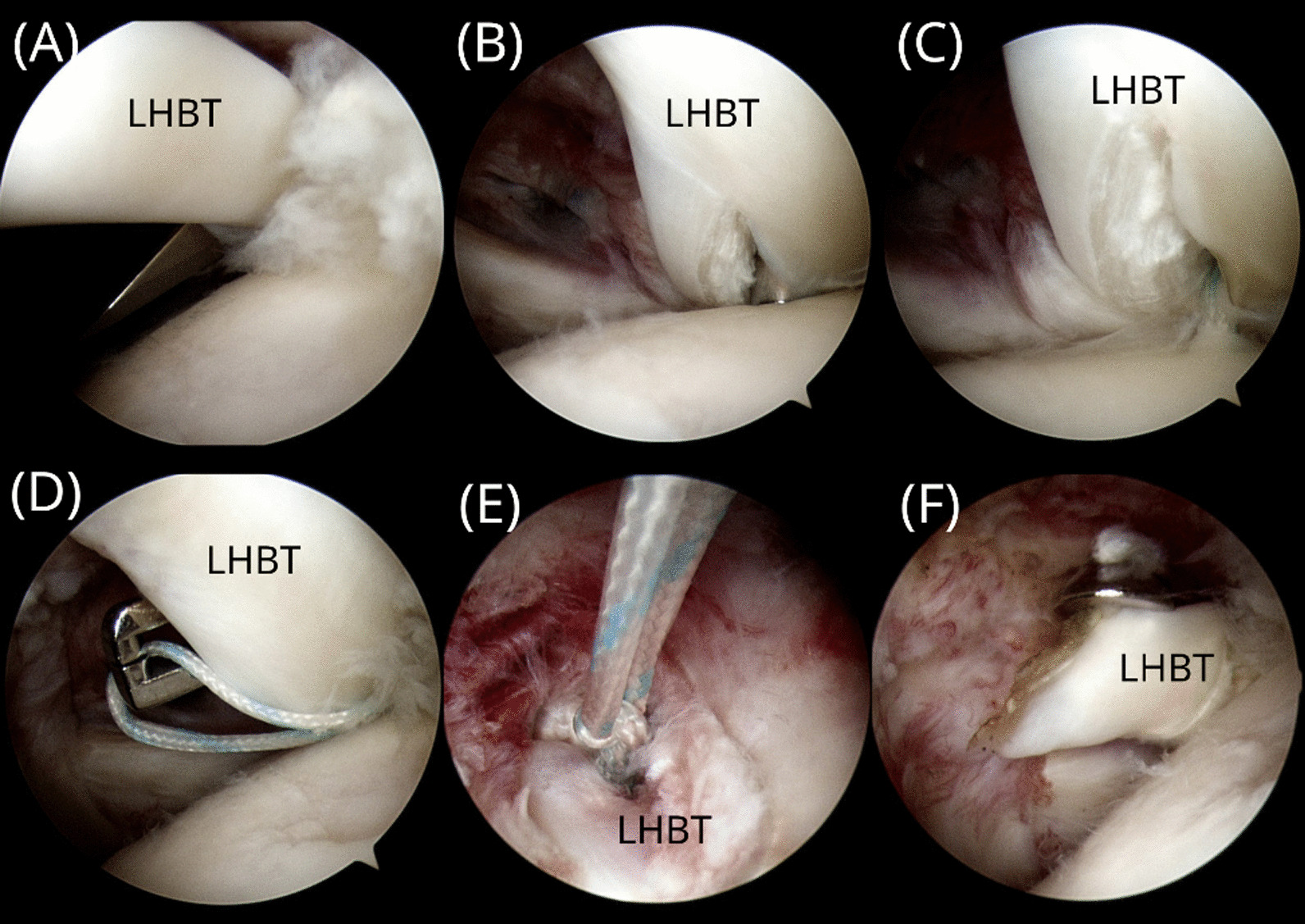
Intraoperative images of the modified transtendinous looped biceps tenodesis (mTLBT) technique. **A** A No. 11 surgical blade is used to make an incision in the long head of biceps tendon (LHBT). **B** A drill guide and an obturator are placed through the LHBT. **C** After pre-drilling, a suture anchor is inserted through the tendon and into the pilot hole. **D** A limb of the suture is passed beneath the tendon for looping the LHBT. **E** The suture limbs are tied on the tendon. **F** Biceps tenotomy is performed, leaving at least 0.5 cm of the tendon stump. **A–D**, **F**: viewed from the standard posterior portal; **E**: viewed from the anterosuperior portal

**Fig. 2:**
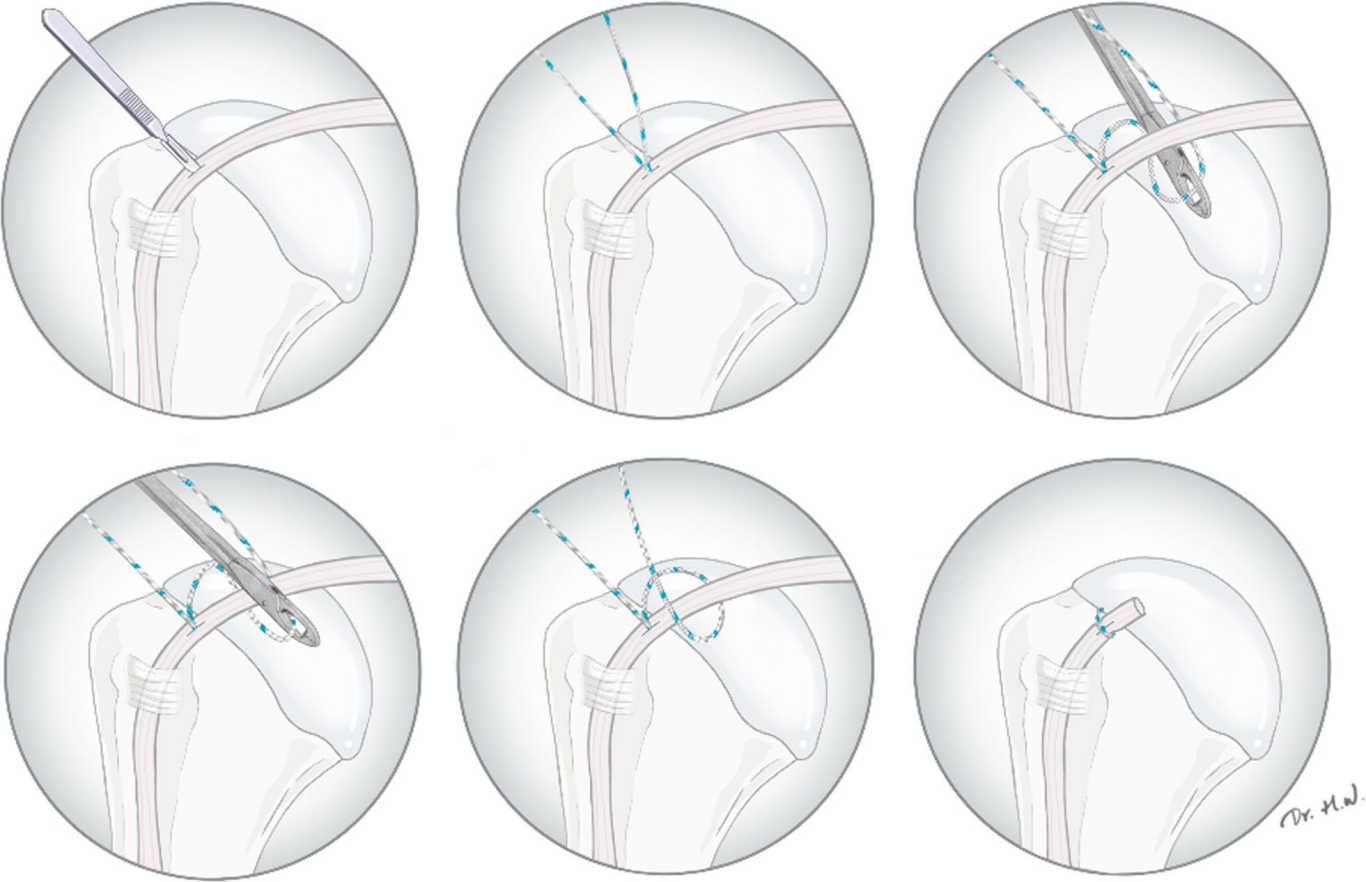
Schematic diagram of the modified transtendinous looped biceps tenodesis (mTLBT) technique

mTLBT was completed through intra-articular portals only. Either all-suture anchors or metallic suture anchors were used for the tenodesis procedures, and the selection of anchors was based on patient preferences. At the beginning of the procedure, a No. 11 surgical blade was used to make a longitudinal incision in the midportion of the LHBT at the proximal inlet of the groove through the anterosuperior portal (Fig. 1A). A drill guide and an obturator were passed through the aforementioned incision in the LHBT to the proximal humerus at the proximal inlet of the groove through the anterosuperior portal (Fig. 1B). A 1.7-mm drill bit was used for all-suture anchors, whereas a 1.6-mm drill bit was used for metallic suture anchors. After pre-drilling, either a 1.7-mm SutureFix Ultra anchor (Smith & Nephew, Andover, MA, USA) or a 2.8-mm TwinFix Ti suture anchor (Smith & Nephew, Andover, MA, USA) was inserted through the tendon and into the pilot hole (Fig. 1C). After anchor insertion, a limb of the suture was passed beneath the tendon to loop the LHBT from the posterior to the anterior (Fig. 1D). The suture limbs were tied to complete the tenodesis construct (Fig. 1E). After tenodesis, biceps tenotomy was performed at the insertion site of the LHBT, preserving at least 0.5 cm of biceps-tendon stump proximal to the tenodesis site (Fig. 1F). A schematic of the mTLBT is illustrated in Fig. 2. After treatment for biceps pathologies, repair of the torn rotator cuff, acromioplasty, or distal clavicular resection were performed arthroscopically, if required.

### Postoperative managements

The same postoperative protocol was used for patients in both groups. In the first 6 weeks postoperatively, a shoulder brace was used for postoperative immobilization; only passive range of motions of the shoulder and elbow were allowed in this period. In postoperative weeks 6–12, patients started active movements of the shoulder and elbow joints; however, elbow flexion was limited to < 1 kg in this period. Three months after surgery, patients could start full weight loading on the shoulder and elbow joints.

### Functional outcome measurements

Clinical outcome data were retrieved through chart review. The shoulder active range of motion, including forward flexion and internal/external rotation with the arm in 90-degree abduction, was assessed preoperatively and at 3, 6, and 12 months postoperatively. Clinical functional outcome scores, including the University of California at Los Angeles (UCLA) and American Shoulder and Elbow Surgeons (ASES) scores [27], were assessed preoperatively and at 6 and 12 months postoperatively. The visual analog scale (VAS) pain score was measured preoperatively and at 1, 2, 3, 6, and 12 months postoperatively. The occurrence of a Popeye deformity (yes or no) was assessed by a single surgeon by observing the cosmetic appearance at 6 months postoperatively. The occurrence of bicipital cramping pain was reported by patients and evaluated using the bicipital squeezing test at 1 month postoperatively. Complications documented in the charts were also recorded.

### Statistical analysis

Statistical analyses were performed using IBM SPSS Statistics version 22 (IBM SPSS Inc., Chicago, IL, USA). Descriptive statistics, including mean and standard deviation, were obtained for both groups. Fisher’s exact test and Chi-square test were used for categorical variables. The Mann–Whitney U test was used to compare nonparametric variables between the two groups. Statistical significance was set at *p* < 0.05.

### Post hoc power analyses

Post hoc power analysis was performed using G*Power Version 3.1.9.7 (Heinrich Heine-University of Dusseldorf, Dusseldorf, Germany) to calculate the achieved power using the available sample sizes and data of Popeye deformity in both groups. The alpha value of the model was determined to be 0.05.

## Results

A total of 15 patients (mean age of 55.4 ± 11.5 years) were enrolled in the mTLBT group, whereas 40 patients (mean age of 64.0 ± 7.0 years) were included in the tenotomy group. In the mTLBT group, metallic anchors were used in seven patients, whereas all-suture anchors were used in eight patients. Demographic information, including sex, laterality, body mass index (BMI), surgical time, and type of biceps pathology, is summarized in Table 1. The mean follow-up period was 12.8 months and 14.1 months for the mTLBT and tenotomy groups, respectively.

**Table 1:** Demographic data and incidence of Popeye deformity in the tenodesis and tenotomy groups

	Tenodesis	Tenotomy	*p* value
Patients, n	15	40	
Male	9 (60.0%)	14 (35.0%)	n.s
Laterality			
Right	8 (53.3%)	20 (50.0%)	n.s
BMI (kg/m^2^)	25.4 ± 3.4	26.9 ± 3.6	n.s
Smoking	4 (26.7%)	8 (20.0%)	n.s
Operative time (min)	41 ± 13	41 ± 17	n.s
Admission days	2.8 ± 1.1	2.8 ± 0.8	n.s
Long head of biceps tendon pathology			
Tear			
None	11 (73.3%)	18 (45.0%)	n.s
Tendinitis	2 (13.3%)	5 (12.5%)	
Tear	2 (13.3%)	17 (42.5%)	
SLAP			
None	0 (0.0%)	5 (12.5%)	n.s
I	3 (20.0%)	11 (27.5%)	
II	12 (80.0%)	23 (57.5%)	
IV	0 (0.0%)	1 (2.5%)	
Instability at bicipital groove	3 (20.0%)	8 (20.0%)	n.s
Subscapularis tears	5 (33.3%)	13 (32.5%)	n.s
Partial supraspinatus tear	4 (26.7%)	15 (37.5%)	n.s
Popeye deformity	2 (13.3%)	21 (52.5%)	0.009*
Biceps cramping pain	3 (20%)	20 (50%)	0.045*

*BMI* body mass index, *SLAP* superior labrum anterior posterior, *n.s.* nonsignificant

*Significant difference (*p* < 0.05) between the tenodesis and tenotomy groups using Fisher’s exact test

The incidence of Popeye deformity and biceps cramping pain was significantly lower in the mTLBT group than in the tenotomy group (*p* = 0.009 and *p* = 0.045, respectively) (Table 1). In terms of shoulder range of motion, patients in the mTLBT group (174 ± 3° flexion, 95 ± 4° external rotation, and 81 ± 12° internal rotation) had results comparable to those of patients in the tenotomy group (171 ± 13° flexion, 92 ± 8° external rotation, and 75 ± 12°internal rotation) at 1 year postoperatively (not significant [n.s.]).

**Fig. 3:**
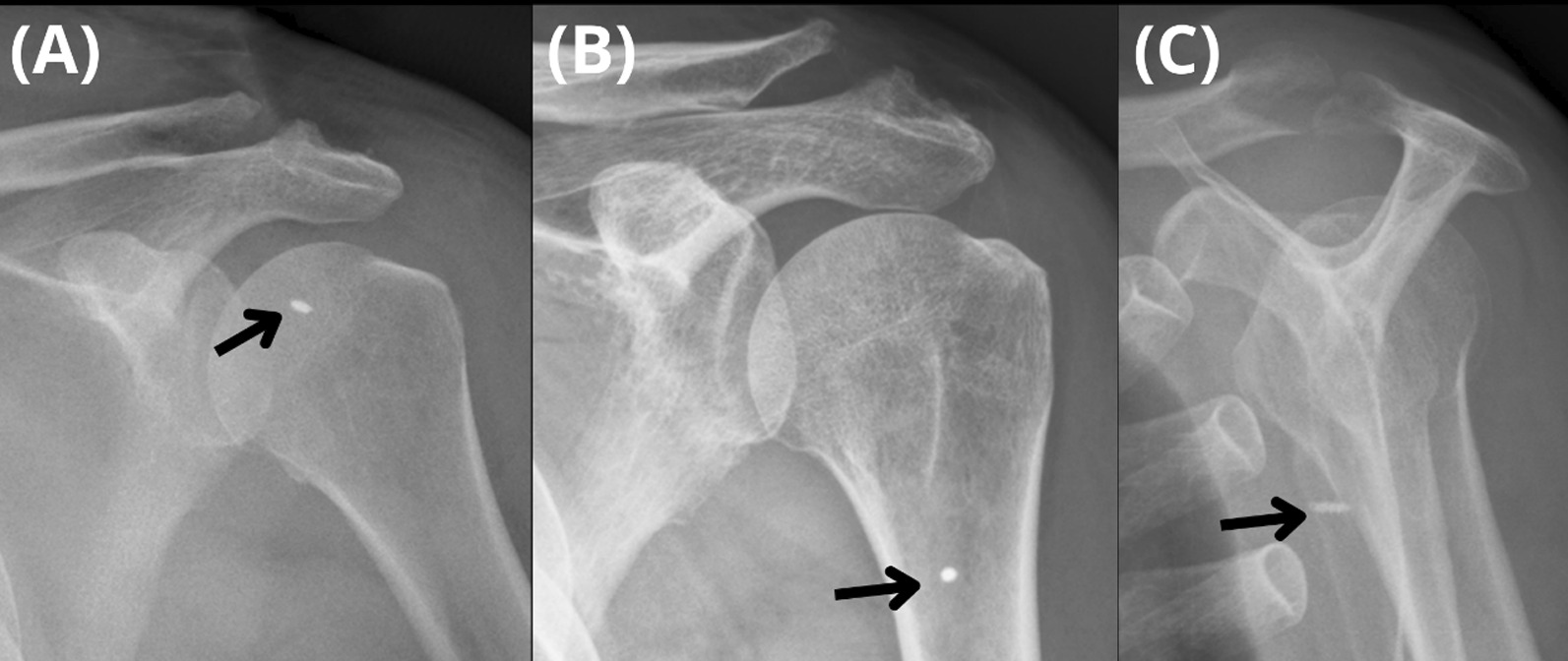
**A** A metallic anchor (arrow) is fixed at the proximal inlet of the bicipital groove. One month after the index surgery, the metallic-anchor pullout is observed on **B** standard anteroposterior and **C** shoulder outlet radiographs

There was no significant difference in VAS pain scores between the two groups in both the preoperative and postoperative periods. In terms of clinical functional outcome scores, the tenodesis group had comparable UCLA and ASES scores to the tenotomy group on the preoperative day and at every time point during postoperative follow-up (Table 2). No postoperative infections were found in either group. One patient in the tenodesis group experienced metallic-anchor pullout at 1 month postoperatively **(**Fig. 3**)**.

**Table 2:** Functional outcomes scores in the tenodesis and tenotomy groups

	Tenodesis (n = 15)	Tenotomy (n = 40)	*p* value
VAS			
Preoperative	4.6 ± 1.1	4.9 ± 1.1	n.s
1 month	2.4 ± 0.9	2.6 ± 1.3	n.s
2 months	1.6 ± 0.8	2.0 ± 1.4	n.s
3 months	1.0 ± 0.7	1.3 ± 1.3	n.s
6 months	0.4 ± 0.5	0.9 ± 1.4	n.s
12 months	0.1 ± 0.3	0.3 ± 0.9	n.s
ASES			
Preoperative	49.2 ± 16.7	46.8 ± 16.6	n.s
6 months	70.2 ± 22.9	72.2 ± 16.0	n.s
12 months	82.0 ± 22.0	78.8 ± 14.7	n.s
UCLA			
Preoperative	17.3 ± 4.5	16.2 ± 4.5	n.s
6 months	26.1 ± 7.9	26.3 ± 3.9	n.s
12 months	29.0 ± 7.4	28.4 ± 3.2	n.s

*n.s.* nonsignificant, *VAS* visual analogue scale, *ASES* American Shoulder and Elbow Surgeons, *UCLA* University of California at Los Angeles

After inputting the actual sample sizes, the proportions of Popeye deformity in both groups, and an α of 0.05 for the post hoc power-analysis model, the calculated achieved power for the study was 81.7%.

## Discussion

The most significant finding in this study was that patients undergoing mTLBT had less biceps cramping pain and less frequent Popeye deformity than those undergoing biceps tenotomy. As biceps tenodesis is becoming a trend for treating biceps pathologies [4], surgical techniques for tenodesis are still developing. The transtendinous technique for suprapectoral biceps tenodesis proposed previously simplifies the suture technique in arthroscopy surgery [16, 25], and our group further modified the surgical technique, performing the procedure through intra-articular portals only. Further, we evaluated the clinical outcomes of this technique and reported reliable results in the tenodesis group compared to the tenotomy group.

The optimal surgical treatment method (tenotomy or tenodesis) for biceps pathology remains controversial. Tenotomy is a simple and low-cost technique with faster recovery, whereas tenodesis can lower the risk of Popeye deformity and theoretically provide better strength [8]. A meta-analysis of level I randomized controlled trials showed that there were differences in the Constant–Murley, VAS, and ASES scores between the tenotomy and tenodesis groups and that patients undergoing biceps tenotomy had a greater risk of cosmetic deformity than those undergoing tenodesis [5]. The findings in the present study are generally consistent with those in the literature [5]. In this study, patients in the tenodesis group had less frequent Popeye deformities and equivalent functional outcome scores at every postoperative follow-up time point compared to those in the tenotomy group. Therefore, the aforementioned findings support the use of the mTLBT technique, which has been newly proposed in this study, in clinical practice.

Although the optimal option for biceps tenodesis remains debatable, transtendinous biceps tenodesis techniques have been promoted as attractive alternatives because the suture-wrapping techniques are simplified and can be easily performed arthroscopically [16, 23, 25]. In this study, the previously described technique [16, 25] was modified, and several advantages were obtained. First, the mTLBT-technique maneuvers were performed in the glenohumeral joint rather than in the subacromial space. Therefore, this modified technique would be especially suitable for patients with partial or no rotator cuff tears because the surgeon does not need to return to the intra-articular space for tenotomy after tendon fixation in the subacromial space [16, 25]. Second, a no. 11 surgical blade was used to make a longitudinal incision in the midportion of the LHBT before pilot-hole drilling for suture anchor insertion. This surgical step is expected to prevent injury to the biceps tendon during drilling, thus reducing tendon rupture after tenodesis. In the present study, two patients had Popeye deformity during follow-up, one of whom encountered a metallic anchor pullout, whereas the other may be related to biceps-tendon rupture or other reasons. As only one patient in the tenodesis group potentially had biceps-tendon rupture, mTLBT could be considered a reliable alternative.

It is well accepted that biceps tenodesis reduces the likelihood of Popeye deformity and biceps cramping pain compared with biceps tenotomy [5, 21]. Similar findings were also observed in this study, in which the frequencies of Popeye deformity and biceps cramping pain were significantly lesser in the tenodesis group. It is worth noting that the incidence of Popeye deformity in the tenotomy group reached 52.5%, which was significantly higher than the mean value of 23% reported in the literature [5, 21]. This phenomenon could be related to whether Popeye deformity was clinician- or self-assessed. Previous studies have indicated that Popeye deformity after biceps long-head surgery was more frequently identified by clinicians than by patients [29, 33]. As Popeye deformity was evaluated by a single orthopedic surgeon in the present study, the high Popeye deformity rate in our patients is not surprising. Interestingly, the body mass index (BMI) is also associated with the occurrence of Popeye deformity [3, 7]. Almeida et al. reported that BMI > 30 kg/m^2^ was related to fewer aesthetic complaints after biceps tenotomy [3], whereas Chiang et al. reported Popeye deformity after biceps tenotomy occurred more frequently in patients with BMIs < 27 kg/m^2^ [7]. In this study, the mean BMI in the tenotomy group was < 27 kg/m^2^, which potentially contributed to the high incidence of Popeye deformities.

### Limitations

This study had some limitations. First, the number of patients included in the two groups was unequal. The patient groups were based on the time period. As the COVID-19 outbreak significantly decreased the case volume in arthroscopy surgeries [26], the enrollment of patients in the tenodesis group was restricted. Despite the smaller number of patients in the tenodesis group, post hoc power analysis confirmed the power to be appropriate. Second, two types of anchors were used for modified transtendinous biceps tenodesis. The selection of anchors was based on the patients’ preference, and the medical expenses for the anchors notably affected patient selection [17]. Due to the relatively small sample size in the tenodesis group, subgroup analyses for all-suture anchors and metallic anchors could not be performed. Third, although elbow flexion and forearm supination forces were considered to be greater in the tenodesis group [18], they were not evaluated in the present study.

## Conclusion

mTLBT is an arthroscopic intra-articular top of the groove tenodesis that can be performed completely in the intra-articular space and is especially suitable for patients with intact or partially torn rotator cuffs. This technique is reliable for treating biceps pathologies as it results in similar functional outcome scores, lesser biceps cramping pain, and less frequent Popeye deformity compared to biceps tenotomy.

## Data Availability

The datasets generated and/or analyzed during the current study are not publicly available but are available from the corresponding author on reasonable request.
